# SCMR president's page

**DOI:** 10.1186/1532-429X-11-4

**Published:** 2009-02-27

**Authors:** Christopher M Kramer

**Affiliations:** 1 Departments of Medicine and Radiology, University of Virginia Health System, Charlottesville, VA, USA

## Editorial

The year 2009 has gotten off to a terrific start for SCMR on many fronts. The 2009 Annual Scientific Session was a resounding success based on the quality of the science and attendance. Overall attendance at all of the meetings including the pre-conferences (physician, basic science, and high field) and the technologist sessions was 1074, essentially unchanged from the prior year which is impressive given the state of the world economy. Hats off to Albert de Roos MD, Program Chair, and Andrew Arai MD, Abstract Chair for putting together an outstanding program including over 100 oral abstracts of the highest quality and over 300 poster presentations as well as the invited talks. The venue at the Rosen Shingle Creek in Orlando was attractive and the meeting rooms were ideal for a meeting of our size and thus the SCMR Board is negotiating to hold the 2012 annual meeting in the same location. As you recall, the 2010 meeting will be in Phoenix, Arizona at the Sheraton Downtown Phoenix hotel from January 21–24 and the 2011 meeting held jointly with the CMR Working Group of the European Society of Cardiology will be in Nice, France.

This is an exciting time for CMR from a politico-economic standpoint as well as scientific. In the U.S., significant progress is being made, with the help of our Advocacy colleagues at the Korris Group as well as individual efforts by Edward (Ted) Martin MD and others from the U.S. Chapter, towards getting Center for Medicare Services (CMS) to pay for the 4 flow codes that make up part of the new 8 payment codes that were newly implemented in January 2008. To this point, CMS has withheld payment for billing any of the 4 codes including flow, based on a dated decision from over 20 years ago. We hope to see this change permanently by early 2010. In addition, SCMR will be critically involved in development of new Multi-Modality Appropriate Use Criteria being put together by the American College of Cardiology (ACC) and American College of Radiology (ACR). The CMR-specific Appropriate Use Criteria will be next in line for renewal after the CT criteria are reworked for the first time since their original publication in 2006. Another exciting development is renewed interest at the FDA for rekindling a specific committee entitled Medical Imaging Drugs Advisory Committee (MIDAC) to deal with contrast agents with imaging expertise on the panel. A recent meeting with FDA was attended by board member Mark Fogel MD along with members of the contrast agent industry community.

Other regional chapters are quite lively including the European, Canadian, and Latin American chapters. The European chapter is heavily involved in implementation of a multi-national CMR imaging registry. The Latin American chapter holds its own annual meeting and puts out a semi-annual newsletter. For the first time, SCMR offered a scholarship to each of the regional chapters for a trainee/junior member presenting an abstract to attend the 2009 annual meeting, a new practice that will continue for future annual scientific sessions.

Activity is high within many of the SCMR committees. The Clinical Trials committee headed by Jeanette Schulz-Menger MD is putting together a group to submit a multi-center, international clinical trial grant proposal to the NIH to study CMR as a predictor of outcome after acute MI. Jeanette will be looking for interested participating sites from around the globe. The Education Committee headed by Victor Ferrari MD is busily working on standardizing 50 cases for display on the SCMR website that will correspond closely to SCMR's recently published standardized protocol document as well as the soon-to-be-published standardized reporting document led by Greg Hundley MD. The Publications Committee and Dudley Pennell MD, editor of JCMR, report that our flagship journal is quite healthy and that the CMR community has responded well to the open access journal that began in January 2008. Over 119 papers were submitted to the journal in 2008 and average time from submission to online publication is incredibly short at only 83 days! The Web Committee with web implementation by James Moon MD is always busy. Web traffic is at an all-time high at approximately 500,000 unique hits and 20 gigabytes of downloaded information per month. A wealth of information and downloadable talks as recent as the annual meeting are available on the website. The site is being moved to a new host, Fragment Media, a process that should take place sometime after March 1^st ^of this year. The science committee headed by Frederick Epstein PhD will tackle another review topic for JCMR in the coming year similar to the perfusion paper published in 2008 as well as plan the basic science pre-conference for the 2010 meeting. The technologist committee led by Mercedes Pereyra RT has developed new guidelines for technologist credentialing.

I am happy to report that our society is quite healthy from a financial and membership perspective. Membership is stable at 1685 at last count and our financial standing has never been better. In fact, our total assets at the end of year 2008 are up 17% from prior year, a luxury that few organizations are likely able to boast about given the present world-wide recession. Strategies are being put in place to develop membership in countries that have been underrepresented in SCMR to date, such as France and China. We have recently renewed our 4-year contract with Talley Management Group and are looking forward to continuing our excellent working relationship with Deborah Berkowitz, Executive Director, and Kathy Baumer, Meeting Manager, as well as others behind the scenes at Talley. We also are grateful for the Platinum level support of Philips and Siemens, who have contributed mightily to the growth and success of the society. In addition, we acknowledge the continuing support of GE, Bayer, and more recently Toshiba, whose participation is highly valued.

Lastly, I would be remiss if I didn't thank our outgoing President Charles B. Higgins MD for his outstanding leadership in the past year. Dr. Higgins did a remarkable job piloting the ship on a steady course through the stormy waters of difficult financial times and delicate relationships with the many international interests within our society. The diversity of SCMR that includes cardiologists, radiologists, other physicians, basic scientists, technologists, nurses, and research coordinators from numerous countries around the globe should be a source of pride for our society. I am personally looking forward to leading SCMR to new heights in the coming year and would appreciate receiving your input and comments at any time. Please feel free to drop me an e-mail or phone call whenever you would like.

